# When Hashimoto’s Thyroiditis Masks Biermer’s Disease: A Revealing Case of Autoimmune Polyendocrinopathy

**DOI:** 10.7759/cureus.82880

**Published:** 2025-04-24

**Authors:** Abrar-Ahmad Zulfiqar

**Affiliations:** 1 Geriatrics, University Hospital of Strasbourg, Strasbourg, FRA

**Keywords:** autoimmune polyendocrinopathy, biermer's disease, hashimoto's thyroiditis, screening, vitamin b12

## Abstract

Biermer's disease is an autoimmune disorder characterized by atrophic gastritis leading to vitamin B12 deficiency, often revealed late due to non-specific symptoms. It may be associated with other autoimmune pathologies, notably Hashimoto's thyroiditis, as part of a type 3b autoimmune polyendocrinopathy (AIP). We report the case of a 44-year-old female patient, followed for autoimmune hypothyroidism, in whom vitamin B12 deficiency was detected. The patient showed no suggestive signs such as glossitis, paresthesia, or other neurological abnormalities. Investigations confirmed Biermer's disease, with no neurological or digestive involvement. Treatment with oral vitamin B12 rapidly corrected the deficiency. While intramuscular vitamin B12 is commonly preferred in autoimmune-related deficiencies, oral supplementation was chosen in this case and proved effective, despite not being the standard approach. This case illustrates the value of systematic vitamin B12 screening in patients with autoimmune thyroiditis and, conversely, the need to monitor thyroid function in any patient with Biermer's disease.

## Introduction

Biermer's disease or pernicious anemia is an underdiagnosed pathology due to its non-specific clinical and biological symptoms, such as subacute combined degeneration, macrocytic anemia, and glossitis, making diagnosis difficult and often delayed [1]. It is characterized by an autoimmune atrophic gastritis responsible for vitamin B12 deficiency, which can develop into severe neurological [2-3], hematological [4], or digestive complications [5] if not diagnosed in time. This condition is also frequently associated with other autoimmune diseases, such as Hashimoto's thyroiditis, type 1 diabetes, or Addison's disease, defining a particular clinical entity known as autoimmune polyendocrinopathy [6]. This constellation of immunological pathologies makes the diagnostic approach more complex, requiring increased vigilance on the part of the clinician. Recognition of these associations is essential to establish comprehensive management and prevent long-term complications. Here, we present a case study to illustrate this point, highlighting the importance of medical reasoning in favor of an autoimmune etiology in the presence of even subtle clinical signs.

## Case presentation

A 44-year-old female patient with a medical history of hypothyroidism associated with Hashimoto thyroiditis, known for five years, on levothyroxine 100 µg daily, presented to her general practitioner with unusual asthenia. Clinical examination was unremarkable from a cardiovascular standpoint, pulmonary auscultation was normal, and abdominal palpation was unremarkable. A laboratory work-up was ordered. Results showed normal hemoglobin at 13.2 g/dl, normocytic (mean globular volume: 87 fl), normal platelets at 334 G/l, no abnormal leukocyte count, no inflammatory syndrome, no electrolyte disorders, normal liver function tests, and creatinine clearance at 149 ml/min/1.73 m^2^. Thyroid function tests showed a TSH of 0.186 mIU/l (laboratory standard: 0.3-4.4 mIU/l). The low TSH suggests levothyroxine over-replacement, and the dosage was subsequently adjusted accordingly. Ferritinemia was normal. An additional biological work-up was then prescribed. Serum protein electrophoresis returned normal. However, a significant vitamin B12 deficiency was noted, with a level of less than 100 pg/ml (laboratory standard: 191-663 pg/ml), with no vitamin B9 deficiency. This vitamin B12 deficiency is confirmed by a second sample. The immunothyroid work-up reveals known anti-thyroperoxidase antibody positivity at 8535 U/ml (laboratory standard: <60) and anti-thyroglobulin antibody positivity at 166 U/ml (laboratory standard: <115 U/ml). 

**Figure 1:**
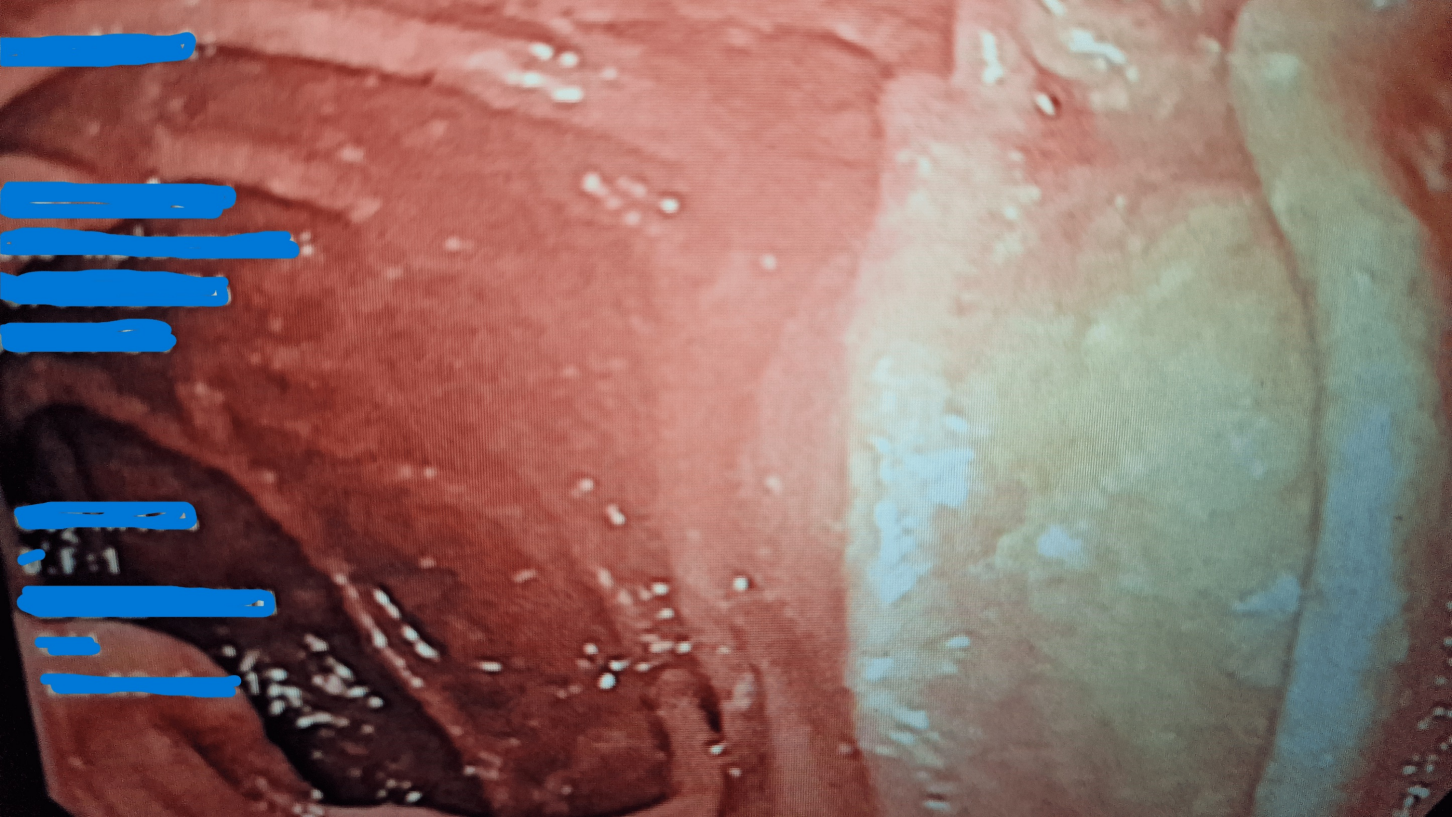
Endoscopic appearance of autoimmune fundic gastric atrophy in pernicious anemia (white area observed in the fundic gastric mucosa)

Clinically, no Hunter's glossitis-type mucosal signs, no digestive signs, and no neurological signs such as paresthesia, sensory, and/or motor deficits were found. As a result of this vitamin B12 deficiency, an etiological work-up revealed positive antiintrinsic factor antibodies at 159 IU/ml (laboratory standard: <1.52 IU/ml), elevated gastrinemia at 658 pg/ml (laboratory standard: 29.4-121 pg/ml), and negative anti-gastric parietal cell antibodies. Although anti-parietal cell antibodies were negative, this result does not exclude autoimmune gastritis, as advanced glandular destruction can lead to seronegativity. In addition, the variability in test sensitivity may contribute to false-negative results. In summary, the patient had a vitamin B12 deficiency secondary to autoimmune destruction of intrinsic factor-producing parietal cells, in connection with probable Biermer's disease. These results are highly specific for pernicious anemia, even in the absence of parietal cell antibodies. As part of the workup for suspected autoimmune gastritis, antral and fundic biopsies by an oesophagogastroduodenal fibroscopy were performed and evaluated using the Updated Sydney Classification. This standardized approach revealed marked fundic atrophy (Figure 1) with intestinal metaplasia, along with a dense lymphoplasmacytic infiltrate in the lamina propria and presence of enterochromaffin-like (ECL) cell hyperplasia and absence of *Helicobacter pylori*. Histological confirmation was supported by the semi-quantitative grading of lesions provided by this classification system.

Overall, the patient presented with a combination of Biermer's disease and Hashimoto's thyroiditis, forming part of a type 3b autoimmune polyendocrinopathy. The patient was tested for autoimmune diabetes, with normal venous glycemia at 1.04 g/l and a normal autoimmune workup. No alopecia or vitiligo (often present in type 3 autoimmune polyendocrinopathy) was observed. In addition, we performed a workup for Addison's disease, which turned out to be negative. Table 1 shows the patient's biological results. On the therapeutic side, immunological vitamin B12 deficiency was treated by taking vitamin B12 orally at 1,000 μg every other day for 10 days, followed by vitamin B12 per os once a month for life. Oral vitamin B12 supplementation was chosen due to the patient's preference to avoid intramuscular injections. This route is also supported by evidence showing adequate absorption even in cases of pernicious anemia when given at high doses. It provided a well-tolerated and effective alternative for long-term management. Biological monitoring of vitamin B12 levels, carried out three months after diagnosis, showed normalization at 225 pg/ml.

**Table 1:** Biological data

Biological parameter	Results	Laboratory standards
Hemoglobin	13.2 g/dl	12-16g/dl
Platelet	334 G/l	150-450 G/l
Mean globular volume	87 fl	82-96 fl
C-related protein	1.4 mg/l	<5 mg/l
Natremia	144 mmol/l	135-145 mmol/l
Kaliemia	4 mmol/l	3.5-5 mmol/l
Aspartate aminotransferase	35 UI/L	15-50 UI/L
Alanine aminotransferase	28 UI/L	15-50 UI/L
Creatinine clearance	149 ml/min/1.73 m2	>90 ml/min/1.73 m2
Thyroid-stimulating hormone (TSH)	0.186 mIU/l	0.4-4 mIU/l
Ferritinemia	70 ng/ml	15-200 ng/ml
Vitamin B12	Less than 100 pg/ml	191-663 pg/ml
Anti-thyroperoxidase antibody	8535 U/ml	<60 U/ml
Anti-thyroglobulin antibody	166 U/ml	<115 U/ml
Anti-intrinsic factor antibodies	159 IU/ml	<1.52 IU/ml
Gastrinemia	658 pg/ml	29.4-121 pg/ml
Venous glycemia	1.04 g/l	0.8-1.26g/l

## Discussion

Biermer's disease is an autoimmune gastric pathology responsible for vitamin B12 deficiency through malabsorption. Vitamin B12 (or cobalamin) deficiency is defined by a serum vitamin B12 concentration below 200 pg/ml, confirmed by a second biological sample [7]. Its prevalence has been estimated at 5% in several recent studies, reaching up to 20% in geriatric patients [7]. There are many causes of deficiency: intake deficiency, Biermer's disease, malabsorption, and, above all, non-dissociation of vitamin B12 from its carrier protein [8]. The latter is linked to the complex metabolism of vitamin B12, which requires the separation of vitamin B12 from dietary proteins and initially involves numerous players: haptocorrin, intrinsic factor, gastric acidity, and pancreatic secretions, all potentially deficient factors. The prevalence of Biermer's disease is 0.1% in the general population and 1.9% in subjects over 65 [8]. It therefore often poses diagnostic and therapeutic problems for the clinician. Physiologically, it is characterized by the presence of autoantibodies directed against parietal cells, whose molecular target is the H+/K+ ATPase [9]. Fundic atrophy is accompanied by reduced gastric acid secretion, reduced intrinsic factor secretion, and vitamin B12 malabsorption, corrected by the addition of intrinsic factor [10]. The gastric enzyme H+/K+ ATPase is the target antigen recognized by anti-gastric parietal cell antibodies [11]. It should be emphasized that the search for Biermer's disease is important, not only because of the consequences of anemia but also because of neurological complications (subacute combined degeneration of the spinal cord) and, above all, a predisposition to gastric tumors of all types, from carcinoid tumors to malignant non-Hodgkin's lymphomas [10]. 

In this case, Biermer's disease was associated with autoimmune hypothyroidism of the Hashimoto's type, and the patient had known Hashimoto's disease for five years. This association appears to be frequent, as seen in a series of 78 patients with Biermer's disease by Markson et al. characterized by antithyroid antibody positivity in 33% of cases [12] and in another study by Doniach et al. involving a series of 100 subjects, with positivity in 47% of cases [13]. Similarly, a retrospective study of 188 patients with Biermer disease showed an association with other autoimmune diseases in 74 of them, with a preferential association with autoimmune thyroiditis [14]. Biermer's disease may be part of a broad group known as autoimmune polyendocrine syndrome (APS), characterized by the coexistence of at least two endocrine deficits linked to an autoimmune mechanism, sometimes with an associated non-endocrine disease [15]. A distinction is made between rare autoimmune polyendocrine syndrome type-1 (APS-1), which affects infants and is characterized by the coexistence of chronic candidiasis, acquired hypoparathyroidism, and peripheral adrenal insufficiency. It is a monogenic syndrome with autosomal recessive inheritance determined by mutations in the AIRE (auto-immune regulator) gene, identified on chromosome 21q22.3 [16]. The AIRE gene is expressed in the thymus, lymph nodes, leukocytes, pancreas, and adrenal cortex. It is a nuclear transcription factor whose mutations are responsible for perturbation of immune tolerance. Autoimmune polyendocrine syndrome type 2 is characterized by the association of primary adrenal insufficiency with thyroid disease (Schmidt's syndrome) and, to a greater or lesser extent, type 1 diabetes (Carpenter's syndrome) in adults [15]. Autoimmune polyendocrine syndrome type 3 is characterized by the master symptom of autoimmune thyroiditis, which may be associated either with autoimmune diabetes (and to a greater or lesser extent with sarcoidosis or celiac disease) defining autoimmune polyendocrine syndrome type 3a; or with Biermer's disease defining autoimmune polyendocrine syndrome type 3b; or with vitiligo and alopecia defining autoimmune polyendocrine syndrome type 3c. It is distinguished from autoimmune polyendocrine syndrome type 2 by the absence of adrenal insufficiency. Very few epidemiological data illustrating type 3 are currently available, given its relatively recent introduction into the classification. Type 3 is thought to be prevalent in women, especially of middle age, despite the fact that it can affect individuals of any age [17]. Sex-based immune modulation, particularly the immunostimulatory effects of estrogen, may contribute to the higher prevalence of autoimmune diseases in females. Estrogen can enhance B-cell activation and antibody production, potentially influencing disease expression and severity. The association observed in the Biermer patient/autoimmune thyroiditis is part of autoimmune polyendocrine syndrome type 3b [17]. Both are polygenic syndromes with autosomal dominant inheritance and incomplete penetrance. The presence of the histocompatibility antigen HLA-DR3 is increased, notably the subtype DR3-DQB1.0102 [18]. This finding supports a role in genetic susceptibility for AIP type 3b, which is often associated with specific HLA haplotypes and clustering of autoimmune conditions, particularly thyroid and gastric disorders.

The major interest of this case report lies in its early detection, with clinical manifestations that can be frustrating or even absent, hence the insidious nature of Biermer disease [10]. While the link between vitamin B12 deficiency and fatigue remains nonspecific, the decision to test and subsequently supplement was guided by both clinical context and supportive biochemical findings, including elevated gastrin levels suggestive of underlying autoimmune gastritis. Although anemia was not present, early-stage B12 deficiency can manifest with subtle or non-hematologic symptoms, and addressing it preemptively may prevent progression to irreversible complications. Thyroid function tests should be carried out systematically in patients with Biermer's disease every year, given the frequent association, as suggested by numerous studies [12,13,19]. Similarly, a patient with autoimmune thyroiditis should have an annual vitamin B12-gastrinemia assay, in both cases on a regular basis for several years, for the earliest possible detection.

## Conclusions

Biermer’s disease often has an insidious course, which can go undetected for several years. The onset of macrocytic anemia should prompt a search for vitamin B12 deficiency, which may be of immunological origin. In cases of known autoimmune thyroiditis, we suggest routine annual vitamin B12 dosage. Early detection through routine B12 monitoring can prevent irreversible neurological and hematological complications, underscoring its clinical relevance in at-risk patients.
